# Centenarian hip fracture patients: a nationwide population-based cohort study of 507 patients

**DOI:** 10.1080/17453674.2019.1602386

**Published:** 2019-04-18

**Authors:** Mathias Mosfeldt, Christian M Madsen, Jes B Lauritzen, Henrik L Jørgensen

**Affiliations:** a Department of Orthopedic Surgery & Department of Molecular Medicine and Surgery, Karolinska University Hospital & Karolinska Institute, Stockholm, Sweden;; b Department of Clinical Biochemistry, Herlev Hospital, University of Copenhagen, Herlev, Denmark;; c Department of Orthopedic Surgery, Bispebjerg Hospital, University of Copenhagen, Copenhagen, Denmark;; d Department of Clinical Biochemistry, Hvidovre Hospital, University of Copenhagen, Hvidovre, Denmark

## Abstract

Background and purpose — Several studies suggest a global increase of centenarians during the 21st century. We describe temporal trends of hip fracture incidence and mortality in this group and compare these patients with a group of younger hip fracture patients with regards to comorbidities and mortality.

Patients and methods — The full study population included all hip fractures that occurred in Denmark (n = 154,047) between 1996 and 2012. Patients aged 100 or above were identified (n = 507) and hip fracture patients between the ages of 70 to 99 years (n = 124,007) were used for comparison. Data were accessed from national registries. Trends in incidence over time were analyzed using a log-linear regression model, mortality was analyzed using the Kaplan–Meier estimator and trends in mortality over time were analyzed using a log-binomial regression model to obtain relative risk estimates.

Results and interpretation — The centenarian patients had fewer comorbidities than the younger comparison group, but mortality was higher at all timepoints. There was no statistically significant change in mortality over time but the incidence of hip fracture among centenarians decreased during the same time period. Our findings describe the characteristics of an emerging group of hip fracture patients and could be of use in the planning of healthcare in the years to come.

As populations are growing increasingly older, new groups of frail patients are emerging. Studies of these patient groups provide insight that might be useful in treatment but also describe characteristics of a population that has survived longer than their peers.

It has been estimated that there will be a global increase from almost half a million centenarians in 2015 to between 13 and 50 million during the 21st century (Robine and Cubaynes 2017). Most currently available studies on centenarian hip fracture patients are based on patient populations that are too small to describe the evolution of the group.

Previous studies on hip fracture patients in general have showed that while the age-adjusted incidence in patients aged 50 and older declined between 1993–1996 and 2001–2005, the incidence rate for patients aged above 90 more than doubled (Bergstrom et al. 2009). We describe temporal trends of incidence and mortality for hip fractures in centenarian patients and compare this group with younger hip fracture patients with regards to comorbidities and mortality in order to gain a greater understanding concerning a group of patients that is expected to increase rapidly during the 21st century. The study was performed using information on the entire Danish population including all hip fractures that occurred over a 17-year period.

## Patients and methods

### Study population

The Danish National Patient Registry was searched for all patients in Denmark aged 18 years or above admitted with a hip fracture (ICD-10 codes DS720 [femoral neck], DS721 [pertrochanteric], and DS722 [subtrochanteric]) from January 1, 1996 to December 31, 2012. The patients were only included once with the first hip fracture that occurred in this period. The full cohort consisted of 154,047 patients. From this cohort we identified 507 patients aged 100 years or above with a hip fracture during this period. We included all 124,007 hip fracture patients between the ages of 70 and 99 years as a comparison group. Aggregated data on annual mortality rates for all individuals aged 99 years or older in the Danish population were available from Statistics Denmark (Danmarks Statistik—statistikbanken.dk) and were included for additional comparison.

### Data sources: national registries

All Danish citizens are registered in the Civil Registration System (CRS) using a unique 10-digit civil registration number (CRN). Demographic information such as vital status and emigration is available from the CRS on everyone residing legally in Denmark. Information from national registries can be linked for the unique individual using the CRN as it is used in all public records. In addition, birth date and sex can be extracted from the CRN.

In Denmark, all contacts and admissions to Danish hospitals are registered in the Danish National Patient Registry (DNPR). This includes data on all somatic hospital admissions dating back to 1977 and has since 1995 also included data on outpatient visits. The DNPR contains information on discharge diagnosis (only 1 is registered) or other secondary diagnostic codes in the form of International Disease Classification (ICD) codes (version 8 and 10). For this study, data from the DNPR were available from 1995 and onwards.

A detailed description of the databases used can be found in a previous article (Madsen et al. 2016). Data from the national registries mentioned was accessed via Statistics Denmark.

### Outcomes and covariates

For the endpoint mortality, date of admission was used as the index date for mortality rates. The patients were followed until death, emigration, or end of follow-up (November 13, 2014), whichever came first. For classification of cause of death in the centenarian cohort, we included information on the underlying cause of death as reported to the Danish Register of Causes of Death (Helweg-Larsen 2011). This information was available only for the 321 deaths occurring in the period 2002 to 2012. Underlying causes of death were grouped according to the overall chapters of the ICD-10 classification as indicated.

Comorbidity was included in the form of the Charlson Comorbidity Index (CCI) and the individual comorbidities making up the index. This was based on data from the DNPR, which records all hospital contacts. If a patient had a hospital contact registered in the DNPR before the time of the fracture with 1 of the ICD-10 codes constituting the comorbidities in the CCI, the patient was listed as having that comorbidity. Hence, a CCI of 0 indicates that the patient has not had a hospital contact registered in the DNPR with any of the comorbidities constituting the CCI. The coding of comorbidities and the ICD-10 codes used for the individual comorbidities were done as described by Quan et al. (2005). All prescription drugs sold in Denmark are registered in the Danish National Prescription Database (Kildemoes et al. 2011). Use of antiosteoporotic medication on admission was defined as retrieving a prescription for antiosteoporotic medication (ATC code: M05B) during the last 6 months before the hip fracture date.

### Statistics

Differences in baseline characteristics were analyzed using Pearson’s chi-squared test for categorical variables and the Mann–Whitney U-test for continuous variables.

Yearly crude absolute incidence rates were calculated as the number of centenarian hip fracture cases each year divided by the total number of centenarians in the Danish population by January 1 each year and expressed as events per 1,000 persons, as done in similar previous publications (Rosengren et al. 2017). This gives a close estimate of the incidence rate obtained by summing up the individual’s person-time at risk, as the competing risk of dying is balanced by the new individuals that enter the group of persons above 100 years of age during the year. Only the first hip fracture during the study period after age 100 was included. Trends in incidence over time were analyzed using a log-linear regression model assuming a Poisson distribution. Including a dispersion parameter did not affect the model fit, and hence there was no overdispersion. The model was constructed as number of cases as a function of calendar year with number of persons at risk each year as offset.

Mortality is given as the proportion of patients dead at certain timepoints and compared in univariate analysis using Pearson’s chi-squared test. Mortality was further analyzed using the Kaplan–Meier estimator and compared using the log-rank test. Median survival times were similarly estimated with the Kaplan–Meier estimator. Trends in 30-day and 1-year mortality over time were analyzed using a log-binomial regression model to obtain relative risk estimates. The model included the dichotomous variable death as a function of calendar year or calendar year, age, and sex. As the aim was to describe change in mortality over time and not to elucidate possible mechanisms or adjust for possible changes, only age and sex were included as additional covariates. This means that the reported estimates could be biased by residual confounding.

2-sided p-values < 0.05 were considered statistically significant. All analyses and data management were conducted using SAS version 9.3 (SAS Institute, Cary, NC, USA) through a secure remote connection provided by Statistics Denmark.

### Ethics, funding, and potential conflicts of interest

The study was approved by the Danish Data Protection Agency (j.nr. 2012-58-0004.) whereas ethical committee approval is not required for this type of observational study according to Danish law. No funding was received for this study and authors have no potential conflicts of interest regarding this investigation.

## Results

### Patient characteristics

#### Comorbidities (Table 1)

A higher percentage of centenarian hip fracture patients had a Charlson Comorbidity Index (CCI) of 0 than was the case for the comparison group of patients between 70 and 99 years of age. Of the centenarians, 68% had a CCI of 0 versus 46% in the comparison group and, similarly, a lower percentage of centenarians had scores of 1, 2, and ≥ 3 as well (17% vs. 22%, 9.1% vs. 16%, and 5.9% vs. 16% respectively), indicating fewer comorbidities among the centenarian hip fracture patients than their younger peers.

#### Mortality

Of the 507 centenarian patients, 484 died during the follow-up period (median: 924 days [385–2121]). Only 23 centenarians were alive at the end of follow-up.

**Figure 1.**
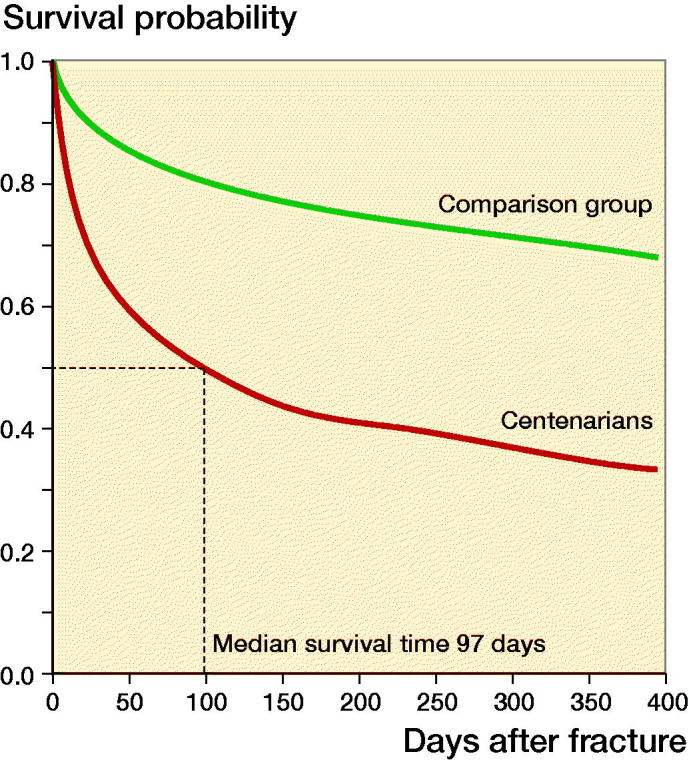
Kaplan–Meier survival curves after hip fracture for centenarians and comparison group of patients between 70 and 99 years of age.

The proportion of centenarian patients that had died within 30 days, 90 days, 1 years, 2 years, and 5 years were 34%, 49%, 66%, 78%, and 94% respectively. Mortality was lower in the comparison group at all timepoints as expected: 11%, 19%, 31%, 42%, and 64% (all p < 0.001). In comparison, for all individuals aged 99 years or above in the Danish population from 1996 to 2012 the annual mortality rate was 48% (Figure 1).

The median estimated survival time was 97 days and 1,029 days in the centenarian and comparison group respectively (logrank p < 0.0001). The difference in median survival between male (70 days) and female (102 days) centenarians was not statistically significant (logrank p = 0.35). The underlying causes of deaths in the 321 centenarians dying in the period 2002 to 2012 are given in Table 2.

**Table 2.** Cause of death for centenarian hip fracture patients occurring in the period 2002–2012 from the Danish Register of Causes of Death. Values are frequency (%)

Cause of death	ICD-10 codes	
Diseases of the circulatory system	I00–I99	94 (29)
External causes of morbidity and mortality (including falls and fractures)	V01–Y98	75 (23)
Ill-defined and unknown causes of mortality	R95–R99	65 (20)
Diseases of the respiratory system	J00–J99	24 (7.5)
Mental and behavioral disorders	F00–F99	22 (6.9)
Diseases of the digestive system	K00–K93	11 (3.4)
Endocrine, nutritional, and metabolic diseases	E00–E90	8 (2.5)
Neoplasms	C00–D48	7 (2.2)
Diseases of the blood and blood-forming organs and certain disorders involving the immune mechanism	D50–D89	5 (1.6)
Diseases of the musculoskeletal system and connective tissue	M00–M99	3 (0.9)
Certain infectious and parasitic diseases	A00–B99	2 (0.6)
Diseases of the skin and subcutaneous tissue	L00–L99	2 (0.6)
Diseases of the genitourinary system	N00–N99	2 (0.6)
Diseases of the nervous system	G00–G99	1 (0.3)

### Temporal trends in incidence and mortality

**Figure 2.**
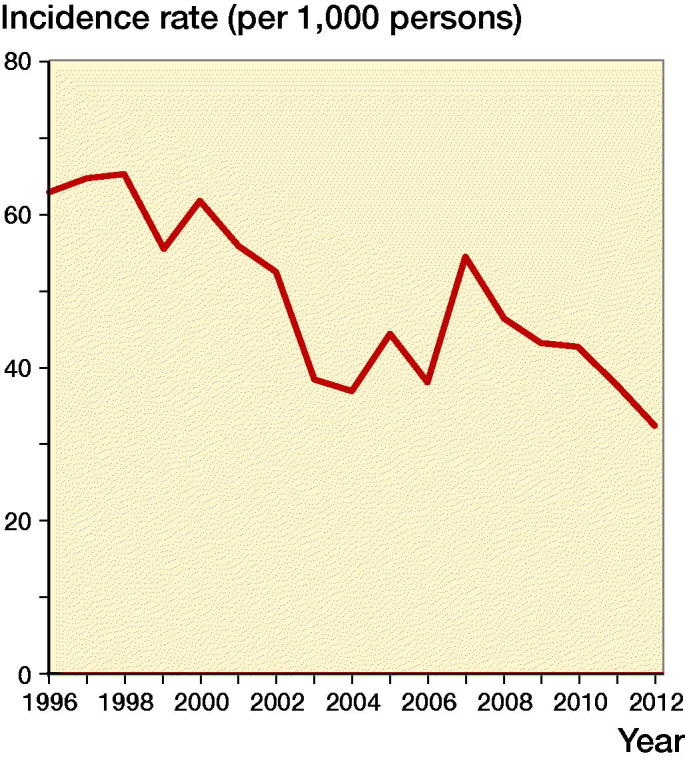
Trend in hip fracture incidence for centenarians.

The annual number of centenarian hip fracture patients in Denmark remained relatively stable and varied between 22 (2003 and 2004) and 39 (2007). The overall incidence rate for the entire period was 47.0/1,000 persons. There was an estimated yearly decrease in the incidence rate of 3.4% (1.7–5.1%, p < 0.001) over the entire study period (Figure 2). The mean age of all centenarians in the Danish population of 101 years was constant from 1996 to 2012.

**Figure 3.**
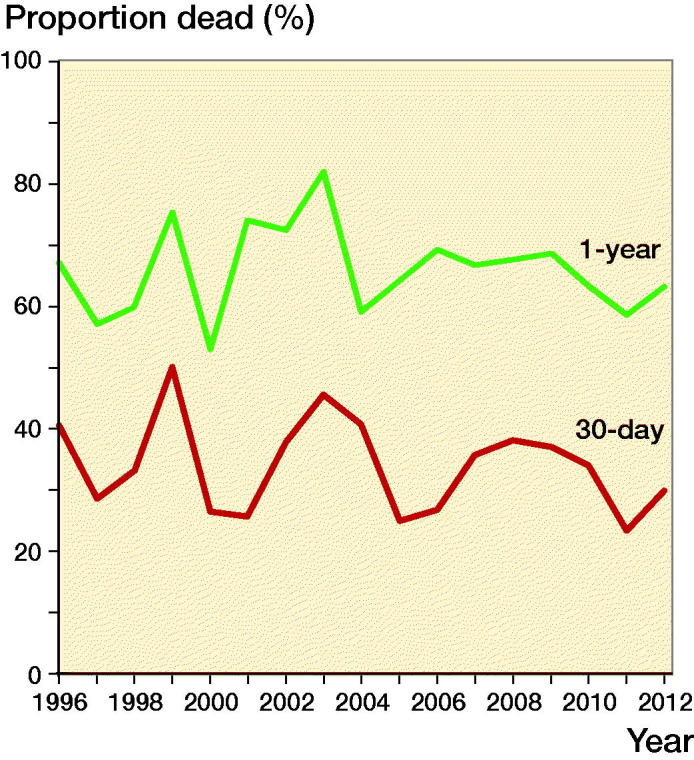
Temporal trends in mortality for centenarians.

There was no statistically significant change in mortality per calendar year for either 30-day (–1.0% [–3.3 to 1.5], p = 0.4) or 1-year (–0.1% [–1.3 to 1.2], p = 0.9) mortality (Figure 3). Adjusting for the possible confounders age and sex had no statistically significant effect on the estimated yearly changes in 30-day (–0.5% [–2.9 to 1.9], p = 0.7) and 1-year (0.2% [–1.1 to 1.4], p = 0.8) mortality. The data were also aggregated into 4, 5-year time periods to account for yearly variations. There was no difference in 30-day and 1-year mortality between any of the time periods. Specifically, for the period 1996–2000 compared with the period 2009–2012 the relative risk was 1.1 [0.82–1.6, p = 0.4] for 30-day mortality and 0.98 [0.82–1.2, p = 0.8] for 1-year mortality.

## Discussion

The incidence of hip fractures in centenarians in Denmark has decreased over the last 2 decades, but the mortality rate following hip fracture among centenarians has remained unchanged aside from negligible yearly variations. In comparison with hip fracture patients between 70 and 99 years of age, the centenarians had fewer recorded comorbidities. Studies of these patient groups provide insight that might be useful in treatment but also describe characteristics of a population that has survived longer than their peers.

Studies on the limits of aging in humans have suggested that the maximal lifespan is approximately 115 years (Dong et al. 2016). In that study, they investigated data from the Human Mortality Database with data from 40 countries and territories and found that while previous increases in life expectancy had been mainly due to decreases in early-life mortality, recent data show a decline in late-life mortality and it would seem that there is still potential for further development of increasing life expectancy.

### Strengths and limitations

The primary limitations of the present study include the lack of information on covariates such as BMI, smoking, frailty, and other variables not available from the national registries. The information on comorbidities is based on hospital visits and hence does not consider comorbidities that have not led to a hospital visit. Therefore, comorbidities solely treated by general practitioners and not leading to hospital contact will not be captured by the Charlson Comorbidity Index based on the registries. Another limitation includes the fact that the included patients were a mix of first and subsequent hip fractures, which could affect the mortality and comparability over time.

The strengths include the above-mentioned population-based design and the use of the high-quality Danish registries. These allow extensive information with high validity on hip fracture diagnoses and mortality. Unlike retrospective chart reviews, the information can be collected in a completely unbiased way from the registries.

### Comparison with literature

Previous studies have shown that the incidence of hip fractures has shifted towards older age (Bergstrom et al. 2009) and that the group of nona- and octogenarians as hip fracture patients is increasing. In some respects, this has created a relatively new population of frail patients with this diagnosis. As is noted in the article by Bergström et al., this makes the population of hip fracture patients even less homogeneous and presents an increasing challenge in management.

As centenarian hip fracture patients are an emerging patient population, there are not many previous studies regarding incidence and outcome; however, it is noted in 1 of the articles that hip fractures were the leading cause of hospital admission in centenarians (Rodriguez-Molinero et al. 2010). It was also noted that centenarians had a 7 times higher incidence of hip fractures than any other age group. The study included all 162 centenarian hip fracture patients in Spain in 2005. The incidence rate was 3.8%, similar to the 4.7% in our study. However, they found a 16% 1-month mortality rate, approximately half of what we found.

Italy and Spain are both among the countries with the highest life expectancies in the world. The centenarian age group in Italy has the highest rate of increase in numbers of the geriatric population. In a cohort of 7,830 centenarians collected between 2004 and 2011, the number of patients who suffered hip fractures was 259, giving an incidence rate of 23/1,000 per year in contrast to the overall incidence rate of 47/1,000 in our study (Mazzola et al. 2016). This is in line with a previous study where the incidence rates have been found to be much higher in Scandinavia than in the rest of Western Europe (Dhanwal et al. 2011). However, only patients who were discharged alive were included in that study and there is no information on in-hospital mortality. Patients were followed up for 2 years and the probability of survival was 32% in the hip fracture cohort compared with 48% in the reference group of centenarians that did not suffer a hip fracture. In comparison, 2-year survival in our study was considerably lower at 23%. The hazard function in the study by Mazzola et al. showed that the greatest risk was 3–4 months postoperatively with an approximation of the trends afterwards in line with previous literature stating that the greatest risk for mortality is in the first 3 months after surgery (Haentjens et al. 2010). In the study by Mazzola et al. (2016), 81% of the centenarians had a Charlson Comorbidity Index of 0 compared with 68% in our study. In a single-center study from Spain on risk factors and mortality rates comparing 33 centenarians with 99 nonagenarian controls (Barcelo et al. 2018), they found that these groups had similar in-hospital outcomes but the centenarians had more postoperative complications. 3-month and 1-year mortality rates were approximately double with 41% and 21% at 3 months, and 62% and 30% for centenarians and nonagenarians respectively. CCI was not different between the groups and while it is difficult to draw conclusions from such a small number of subjects it is noteworthy that the centenarians had fewer readmissions than the nonagenarians after 3 months and after 1 year.

Concerning temporal trends, previous studies have shown regional differences in hip fracture incidence, which in the Western world seems to have stabilized and decreased during the latest decades whereas it has increased in the developing world (Morin et al. 2013). In Denmark specifically, the incidence of hip fractures declined by about 20% between 1997 and 2006 (Abrahamsen et al. 2010). Our study period coincides with the widespread introduction of antiosteoporotic treatment and an increased focus on fracture prevention in the elderly, which may contribute to the declining incidence rates. Improvements in nutrition might also influence declining rates of hip fracture incidence during the study period. Also during the study period, there has been a decrease in smoking habits in Denmark (Ng et al. 2014), which might contribute positively to bone health.

Furthermore, and also in line with our results, showing no change in mortality rates over time, a Swedish population-based study found that mortality following hip fracture had remained unchanged from 1995 to 2012 (Karampampa et al. 2015). This also included the age group 95+, but no specific information was given for those aged 100 years or above.

### Conclusions and implications

Our study is one of the largest conducted to date concerning centenarian hip fracture patients. Mortality was higher in centenarians than in younger patients but seemed stable over time aside from slight variations from year to year. There was a yearly decrease in incidence rates for centenarian hip fractures over the entire study period. The centenarians in our study seemed to have fewer comorbidities than their younger counterparts.

Our findings support the “compression of morbidity” hypothesis of aging (Fries 1980) for this patient group, as it seems that many have reached an advanced age while having few or no comorbidities. This would suggest that for many of the centenarians in this study, onset of morbidity occurred late and was relatively short before their death, occurring not long after their hip fracture. This could be an important consideration in the planning of healthcare for the coming decades as patients are expected to reach increasingly higher ages, but the patients in this study who lived for more than 100 years had fewer registered diagnoses from hospital contacts than the younger hip fracture patients.

**Table 1.** Basic characteristics for centenarians and comparison group. Values are frequency (%) unless otherwise specified

	Comparison		
	Centenarians	group	p-value
n	507	124,007	NA
Women	431 (85.0)	91,439 (73.7)	< 0.001
Age (years), median (range)	101 (100–111)	83 (70–99)	NA
Charlson Comorbidity Index, median (range)	0 (0–7)	1 (0–19)	< 0.001
Charlson Comorbidity Index			< 0.001
0	344 (67.9)	56,451 (45.5)	
1	87 (17.2)	27,421 (22.1)	
2	46 (9.1)	19,796 (16.0)	
3	30 (5.9)	20,339 (16.4)	
Renal disease	2 (0.4)	2,192 (1.8)	0.01
Liver disease, mild	1 (0.2)	873 (0.7)	0.3
Liver disease, severe	0	217 (0.2)	1.0
Congestive heart failure	85 (16.8)	27,568 (22.2)	0.003
Myocardial infarction	21 (4.1)	7,622 (6.2)	0.06
DM wIith complications	3 (0.6)	2,918 (2.4)	0.005
DM without complications	8 (1.6)	8,567 (6.9)	< 0.001
Cerebrovascular disease	50 (9.9)	18,322 (14.8)	0.002
Peripheral vascular disease	6 (1.2)	7,480 (6.0)	< 0.001
Pulmonary disease	12 (2.4)	12,183 (9.8)	< 0.001
Ulcer disease	18 (3.6)	7,074 (5.7)	0.04
Malignancy	22 (4.3)	13,836 (11.2)	< 0.001
Solid metastatic tumor	1 (0.2)	1,488 (1.2)	0.04
Rheumatic disorders	6 (1.2)	4,670 (3.8)	0.002
Dementia	16 (3.2)	10,641 (8.6)	< 0.001
Paralysis	0	335 (0.3)	0.7
Fracture type			0.06
Femoral neck	273 (53.9)	73,125 (59.0)	
Pertrochanteric	203 (40.0)	43,569 (35.1)	
Subtrochanteric	31 (6.1)	7,299 (5.9)	
Use of antiosteoporotics	6 (1.2)	7,921 (6.4)	< 0.001

DM: Diabetes mellitus

## References

[CIT0001] Abrahamsen B, Vestergaard P Declining incidence of hip fractures and the extent of use of anti-osteoporotic therapy in Denmark 1997–2006. Osteoporos Int 2010; 21(3): 373–80. 19436931 10.1007/s00198-009-0957-3

[CIT0002] Barcelo M, Francia E, Romero C, Ruiz D, Casademont J, Torres OH. Hip fractures in the oldest old: comparative study of centenarians and nonagenarians and mortality risk factors. Injury 2018; 49(12): 2198–202. 30274759 10.1016/j.injury.2018.09.043

[CIT0003] Bergstrom U, Jonsson H, Gustafson Y, Pettersson U, Stenlund H, Svensson O The hip fracture incidence curve is shifting to the right. Acta Orthop 2009; 80(5): 520–4. 19916682 10.3109/17453670903278282 PMC2823331

[CIT0004] Dhanwal D K, Dennison E M, Harvey N C, Cooper C Epidemiology of hip fracture: worldwide geographic variation. Indian J Orthop 2011; 45(1): 15–22. 21221218 10.4103/0019-5413.73656 PMC3004072

[CIT0005] Dong X, Milholland B, Vijg J Evidence for a limit to human lifespan. Nature 2016; 538(7624): 257–9. 27706136 10.1038/nature19793 PMC11673931

[CIT0006] Fries J F Aging, natural death, and the compression of morbidity. N Engl J Med 1980; 303(3): 130–5. 7383070 10.1056/NEJM198007173030304

[CIT0007] Haentjens P, Magaziner J, Colón-Emeric C S, Vanderschueren D, Milisen K, Velkeniers B, Boonen S Meta-analysis: excess mortality after hip fracture among older women and men. Ann Intern Med 2010; 152(6): 380–90. 20231569 10.1059/0003-4819-152-6-201003160-00008 PMC3010729

[CIT0008] Helweg-Larsen K The Danish Register of Causes of Death. Scand J Public Health 2011; 39(7 Suppl): 26–9. 10.1177/1403494811399958 21775346

[CIT0009] Karampampa K, Ahlbom A, Michaelsson K, Andersson T, Drefahl S, Modig K Declining incidence trends for hip fractures have not been accompanied by improvements in lifetime risk or post-fracture survival: a nationwide study of the Swedish population 60 years and older. Bone 2015; 78: 55–61. 25933944 10.1016/j.bone.2015.04.032

[CIT0010] Kildemoes H W, Sorensen H T, Hallas J The Danish National Prescription Registry. Scand J Public Health 2011; 39(7 Suppl): 38–41. 21775349 10.1177/1403494810394717

[CIT0011] Madsen C M, Jantzen C, Lauritzen J B, Abrahamsen B, Jorgensen H L Temporal trends in the use of antithrombotics at admission. Acta Orthop 2016; 87(4): 368–73. 27301556 10.1080/17453674.2016.1195662 PMC4967279

[CIT0012] Mazzola P, Rea F, Merlino L, Bellelli G, Dubner L, Corrao G, Pasinetti G M, Annoni G Hip fracture surgery and survival in centenarians. J Gerontol A Biol Sci Med Sci 2016; 71(11): 1514–8. 26883679 10.1093/gerona/glw016

[CIT0013] Morin S N, Lix L M, Majumdar S R, Leslie W D Temporal trends in the incidence of osteoporotic fractures. Curr Osteoporos Rep 2013; 11(4): 263–9. 24078485 10.1007/s11914-013-0168-x

[CIT0014] Ng M, Freeman M K, Fleming T D, Robinson M, Dwyer-Lindgren L, Thomson B, Wollum A, Sanman E, Wulf S, Lopez A D, Murray C J, Gakidou E Smoking prevalence and cigarette consumption in 187 countries, 1980–2012. JAMA 2014; 311(2): 183–92. 24399557 10.1001/jama.2013.284692

[CIT0015] Quan H, Sundararajan V, Halfon P, Fong A, Burnand B, Luthi J C, Saunders L D, Beck C A, Feasby T E, Ghali W A Coding algorithms for defining comorbidities in ICD-9-CM and ICD-10 administrative data. Med Care 2005; 43(11): 1130–9. 16224307 10.1097/01.mlr.0000182534.19832.83

[CIT0016] Robine J M, Cubaynes S Worldwide demography of centenarians. Mech Ageing Dev 2017; 165(Pt B): 59–67. 28315698 10.1016/j.mad.2017.03.004

[CIT0017] Rodriguez-Molinero A, Yuste A, Banegas J R High incidence of hip fracture in Spanish centenarians. J Am Geriatr Soc 2010; 58(2): 403–5. 10.1111/j.1532-5415.2009.02706.x 20370877

[CIT0018] Rosengren B E, Bjork J, Cooper C, Abrahamsen B Recent hip fracture trends in Sweden and Denmark with age-period-cohort effects. Osteoporos Int 2017; 28(1): 139–49. 27647528 10.1007/s00198-016-3768-3 PMC5206266

